# Synthesis of Poly(ethylene furanoate) Based Nanocomposites by In Situ Polymerization with Enhanced Antibacterial Properties for Food Packaging Applications

**DOI:** 10.3390/polym15234502

**Published:** 2023-11-23

**Authors:** Johan Stanley, Eleftheria Xanthopoulou, Matjaž Finšgar, Lidija Fras Zemljič, Panagiotis A. Klonos, Apostolos Kyritsis, Savvas Koltsakidis, Dimitrios Tzetzis, Dimitra A. Lambropoulou, Diana Baciu, Theodore A. Steriotis, Georgia Charalambopoulou, Dimitrios N. Bikiaris

**Affiliations:** 1 Laboratory of Chemistry and Technology of Polymers and Colors, Department of Chemistry, Aristotle University of Thessaloniki, GR-54124 Thessaloniki, Greece; johansta@chem.auth.gr (J.S.); elefthxanthopoulou@gmail.com (E.X.); 2 Faculty of Chemistry and Chemical Engineering, University of Maribor, SI-2000 Maribor, Slovenia; matjaz.finsgar@um.si; 3 Faculty of Mechanical Engineering, University of Maribor, SI-2000 Maribor, Slovenia; lidija.fras@um.si; 4 Department of Physics, National Technical University of Athens, Zografou Campus, GR-15780 Athens, Greece; pklonos@central.ntua.gr (P.A.K.); akyrits@central.ntua.gr (A.K.); 5 Digital Manufacturing and Materials Characterization Laboratory, International Hellenic University, GR-57001 Thessaloniki, Greece; skoltsakidis@ihu.edu.gr (S.K.); d.tzetzis@ihu.edu.gr (D.T.); 6 Laboratory of Environmental Pollution Control, Department of Chemistry, Aristotle University of Thessaloniki, GR-54124 Thessaloniki, Greece; dlambro@chem.auth.gr; 7 Center for Interdisciplinary Research and Innovation (CIRI-AUTH), Balkan Center, GR-57001 Thessaloniki, Greece; 8 National Center for Scientific Research “Demokritos”, GR-15341 Ag. Paraskevi Attikis, Greece; dianabaciuro@yahoo.com (D.B.); t.steriotis@inn.demokritos.gr (T.A.S.); gchar@ipta.demokritos.gr (G.C.)

**Keywords:** biobased polymers, poly(ethylene furanoate), nanoparticles, nanocomposites, antimicrobial properties

## Abstract

Poly(ethylene 2,5-furandicarboxylate) (PEF)-based nanocomposites containing Ce–bioglass, ZnO, and ZrO_2_ nanoparticles were synthesized via in situ polymerization, targeting food packaging applications. The nanocomposites were thoroughly characterized, combining a range of techniques. The successful polymerization was confirmed using attenuated total reflectance Fourier-transform infrared (ATR-FTIR) spectroscopy, and the molecular weight values were determined indirectly by applying intrinsic viscosity measurements. The nanocomposites’ structure was investigated by depth profiling using time-of-flight secondary ion mass spectrometry (ToF-SIMS), while color measurements showed a low-to-moderate increase in the color concentration of all the nanocomposites compared to neat PEF. The thermal properties and crystallinity behavior of the synthesized materials were also examined. The neat PEF and PEF-based nanocomposites show a crystalline fraction of 0–5%, and annealed samples of both PEF and PEF-based nanocomposites exhibit a crystallinity above 20%. Furthermore, scanning electron microscopy (SEM) micrographs revealed that active agent nanoparticles are well dispersed in the PEF matrix. Contact angle measurements showed that incorporating nanoparticles into the PEF matrix significantly reduces the wetting angle due to increased roughness and introduction of the polar -OH groups. Antimicrobial studies indicated a significant increase in inhibition of bacterial strains of about 9–22% for Gram-positive bacterial strains and 5–16% for Gram-negative bacterial strains in PEF nanocomposite films, respectively. Finally, nanoindentation tests showed that the ZnO-based nanocomposite exhibits improved hardness and elastic modulus values compared to neat PEF.

## 1. Introduction

Active and intelligent packaging to guarantee food safety and quality and extend the shelf life of food products has attracted significant attention in recent years. It has been a challenge for food industries to satisfy consumers’ demand for fresh and delicious food products with reliable quality and safety [1]. Active packaging systems include antimicrobial packaging, antioxidant packaging, ethylene moisture absorbers, carbon dioxide emitters, and freshness indicators [2,3]. These systems are typically prepared by incorporating different active agents (e.g., nanoparticles, biomolecules) into packaging materials, and their function involves the release of the active agents into the surrounding environment or the sorption of food-derived chemicals. One of the most important safety aspects of food packaging is to improve the microbial shelf life of food products. Microbes such as bacteria and viruses in the environment may cause mild-to-deadly diseases. These microbes can also be transmitted through infected objects, contaminated surfaces, water, or food. Since the COVID-19 pandemic, intensive research has been devoted to developing antimicrobial polymer packaging for agriculture, food, and cosmetics applications [4]. In addition to antimicrobial properties, the packaging materials should possess excellent mechanical, optical, and thermal properties to protect food products against vibration, tension, and compression [5].

Biobased polymeric materials derived from renewable resources, such as biomass, are promoted as an alternative to their petroleum-derived counterparts for safer, sustainable, and green packaging. The actual application of biobased polymers as food packaging materials, involving their composites, is to satisfy requirements like mechanical, permeability, and antimicrobial properties [6]. Poly(ethylene 2,5-furandicarboxylate) (PEF) is a biobased polyester that has received attention from the industry and academia thanks to its superior thermal properties, higher oxygen and carbon dioxide, and water barrier properties, as it can be used as a 100% biobased alternative to the petrochemically derived poly(ethylene terephthalate) (PET) [7]. Compared to PET, the newly synthesized PEF bottles have shown 11 times better O_2_ and 19 times better CO_2_ barrier function, 1.6 times higher tensile modulus value, approximately 70% less CO_2_ emission, and approximately 65% less nonrenewable energy for production [8]. The main concern for using PEF in safer food packaging is its poor antimicrobial properties, a problem that can be solved by incorporating active ingredients. Antimicrobial materials used in food packaging involve organic/inorganic nanomaterials, bacteriocins or enzymes of bacterial origin, extracts, essential oils, and other natural sources. The edible oils derived from the plants are used in flavoring additives in foods and preventing food spoilage [9]. Incorporating inorganic nanoparticles into a polymeric matrix has been widely used due to their potential antimicrobial activity and contribution to enhancing the mechanical and barrier properties of the respective films [10]. However, safety aspects associated with the use of nanoparticles in food packaging should also be considered. Generally, nanoparticles on the surface of the packaging materials do not cause adverse effects on consumers’ health, but their transfer or integration into food products may be problematic. Within this frame, the European Commission has defined a specific release limit (SRL) for authorized nanoparticles (e.g., zinc = 5 mg/kg food and zirconium = 2 mg/kg food) to determine the release of substances from the polymeric materials used for food packaging applications [11].

Nevertheless, not all types of nanoparticles possess antimicrobial activity due to their different chemical nature. Among them, metal/metal oxide inorganic nanoparticles are extensively used as nanofillers in food packaging systems thanks to their remarkable antimicrobial potential. Specifically, the most commonly used nanoparticles in such applications are silver (Ag), zinc oxide (ZnO), copper oxides (Cu, CuO, CuS), magnesium oxide (MgO), selenium (Se), palladium (Pd), iron (Fe), silicon dioxide (SiO_2_), and titanium dioxide (TiO_2_). Atta et al. developed a bacterial–cellulose (BC)–Ag nanocomposite film as a biocompatible and edible fruit-coating biomaterial to protect and extend the shelf of various food products [12]. Recently, some studies have been carried out to improve the antimicrobial activity of PEF by incorporating ZnO nanoparticles (ZnO NPs) using the solvent casting method in the polymer matrix and fabrication of gallium-doped ZnO (GZO) on the surface of PEF films [10,13].

ZnO is a promising antimicrobial agent for active packaging applications due to its white appearance, high versatility, and ultraviolet light-blocking properties. The use of ZnO as an antimicrobial agent started in 1995, and it has been recognized as a safe food contact material by the US Food and Drug Administration (FDA). Recent studies have found that beyond its antimicrobial activity, it can also improve biopolymer mechanical, thermal, and barrier properties [14].

There have been very few studies examining the use of zirconium dioxide nanoparticles (ZrO_2_NPs) in food packaging applications [15]. ZrO_2_ nanoparticles exhibit antimicrobial, antifungal, and antioxidant properties. At the same time, it is worth mentioning that over the years, ZrO_2_ has been used in various engineering applications due to its high chemical stability, excellent mechanical strength, high melting point, better resistance to fracture, photostability, hydrophobicity, ion conductivity, and bio-inertness [16].

Nevertheless, other types of inorganic materials with significant antimicrobial efficacy, such as bioglasses, have not been extensively examined as components in the food packaging sector. Bioglasses are a series of specially designed, surface-reactive silica-based glass-ceramic porous materials primarily comprising a three-dimensional SiO_2_ network that can be modified by incorporating metal oxides such as Na_2_O, CaO, and P_2_O_5_. They are extensively used for orthopedic and dental implants by tuning their composition, and it is possible to induce specific functionality and biological responses ranging from stimulating tissue regeneration and repair to preventing bacterial infections. Among others, it has been reported that the presence of Ce ions in the bioglass composition has been associated with enhanced antimicrobial activity [17]. Cerium oxide nanoparticles possess higher activities against many Gram-positive and Gram-negative bacteria, including a few multidrug-resistant pathogenic species [18].

Herein, the synthesis of PEF-based nanocomposites incorporating inorganic additives via in situ polymerization is reported for the first time. Specifically, a small amount (1 wt.%) of each nanoparticle, such as ZnO, ZrO_2_, and bioglass doped with Ce, was incorporated into the reaction mixture. For this purpose, a Ce mesoporous bioactive glass system was prepared based on a facile PEG–CTAB-assisted sol–gel method using poly(ethylene glycol) (PEG) and cetyltrimethylammonium bromide (CTAB) as nonionic cosurfactant and cationic surfactant, respectively. Both the synthesized and purchased nanoparticles were less than 100 nm. All produced PEF-based nanocomposites have been extensively characterized regarding their intrinsic viscosity/molecular weight values as well as their structural, physicochemical, and thermal properties and performance, combining a range of methods (crystallinity studies, microscopy analysis, spectroscopy measurements, water contact angle (CA) measurements, nanoindentation tests, antimicrobial studies).

## 2. Materials and Methods

### 2.1. Materials

The 2,5-furan dicarboxylic acid (BioFDCA X000230-2003) was purchased from Corbion (Gorinchem, the Netherlands). Ethylene glycol (anhydrous, 99.8%) and antimony trioxide were purchased from Aldrich Co. (London, UK). ZnO and ZrO_2_ (<100 nm), which were used as active agents, were purchased from Alfa Aesar (Ward Hill, MA, USA) and Sigma Aldrich (St. Louis, MO, USA), respectively. Poly(ethylene glycol) (PEG) particles (average Mn (10.000)), tetraethyl orthosilicate (TEOS) (98%), triethyl phosphate (≥99.8%), strontium nitrate Sr(NO_3_)_2_ (ACS reagent, ≥99%), cerium(III) nitrate hexahydrate (99% trace metals basis), magnesium chloride hexahydrate Cl_2_Mg·6H_2_O (99–100%), cetyltrimethylammonium bromide (labeled as CTAB) and ethanol were all purchased from Sigma Aldrich Chemical Company (St. Louis, MO, USA). Ammonium hydroxide (30 wt.% NH_3_ in water) was purchased from Panreac. All other materials and solvents used were of analytical grade.

#### Synthesis of Mesoporous Bioglass (Ce–Bioglass)

For the preparation of the mesoporous bioglass (denoted as Ce–bioglass) with a composition of 78.5% SiO_2_, 10% SrO, 10% P_2_O_5_, 0.5% MgO, and 1% CeO_2_ (in mol), 0.66 g CTAB and 0.6 g PEG particles were dissolved in 1000 mL distilled water and 26 mL ammonium hydroxide under vigorous stirring. After 15 min, 2.96 mL TEOS, 0.28 mL triethyl phosphate, 0.35 g Sr (NO_3_)_2_, 0.017 g Cl_2_Mg*6H_2_O and 0.073 g cerium (III) nitrate hexahydrate were added to the mixture and stirred for 3 h at room temperature. The products were collected by centrifugation, washed several times with distilled water and ethanol, and dried in a Petri dish at 60 °C using a hot plate. Finally, the obtained white powder was calcined in air at 600 °C for 5 h with a heating rate of 5 °C/min.

### 2.2. Synthesis of Polyester Using 2,5-Furandicarboxylic Acid and Respective Nanocomposites

Poly(ethylene 2,5-furandiacarboxylate) (PEF) was synthesized via a two-stage polycondensation method. In the first step (esterification), FDCA and EG were utilized in a 1:2.1 molar ratio, and 1 wt.% of each active agent was incorporated into the reaction flask, as listed in Table 1. Initially, the flask was evacuated and filled with nitrogen several times to eliminate oxygen. The reaction mixture was heated at 170 °C for 30 min, 190 °C for 1 h, 200 °C for 30 min, and 210 °C for 30 min under nitrogen and a stirring speed of 200 rpm. After the completion of the first step, an antimony trioxide (Sb_2_O_3_) (300 ppm) catalyst was added to the reaction flask, and a vacuum (5.0 Pa) was applied slowly for 15 min to initiate the polycondensation process. Furthermore, the temperature was progressively increased to 250 °C. The reaction mixture was heated at 250 °C and 260 °C for 2 h each time. At the same time, the stirring speed decreased (100–70–50 rpm) to avoid high shear stress while the viscosity increased. Finally, the samples were retrieved from the reaction mixture, milled, and washed with methanol to remove unreacted substances.

### 2.3. Characterization

#### 2.3.1. Intrinsic Viscosity Measurement

Intrinsic viscosity ([*ղ*]) measurements were performed using an Ubbelohde viscometer at 30 °C in a mixture of phenol/1,1,2,2-tetrachloroethane at a ratio of (60/40, *w*/*w*). Sample concentrations of 1% (*w*/*v*) were used. The [*ղ*] value of each sample was calculated using the following Solomon–Ciuta equation (1):

(1) $$\left[\eta \right]=\frac{{[2\{\frac{t}{t_{0}}-\ln\left(\frac{t}{t_{0}}\right)-1\}]}^{\frac{1}{2}}}{c}$$

where *c* is the concentration of the solution, *t*_0_ is the flow time of pure solvent, and *t* is the flow time of solution. The measurements were performed in triplicate, and the mean value was calculated.

The average molecular weight ($M\bar{n}$) of the samples was determined using the following Berkowitz Equation (2):

(2) $$M\bar{n}=3.29\times 10^{4}{\left[\eta \right]}^{1.54}$$

#### 2.3.2. Color Measurements

A Datacolor Spectraflash SF600 plus CT UV reflectance colorimeter (Datacolor, Marl, Germany) was used to measure color using the D65 illuminant and a 10° standard observer by excluding the UV component and including the specular component. Five measurements were taken in each case using a unique holder (Datacolor), and the mean values were computed. The color of the PEF films was investigated according to the CIEL*a*b* color system. The L* axis measures luminosity or lightness ranging from 0 (black) to 100 (white), the a* coordinate measures redness when a is positive or greenness when a is negative, and the b* coordinate measures yellowness when b is positive or blueness when b is negative, C* represents chroma and H* represents hue angle. The K/S fraction was calculated to assess the concentration of the color on the PEF films [19].

#### 2.3.3. Attenuated Total Reflectance Fourier-Transform Infrared (ATR-FTIR) Spectroscopy

ATR-FTIR spectra of the samples were recorded utilizing an IRTracer-100 (Shimadzu, Japan) equipped with a QATR™ 10 single-reflection ATR module with a diamond crystal. The spectra were collected from 450 to 4000 cm^−1^ at a resolution of 2 cm^−1^ (total of 16 scans).

#### 2.3.4. Time-of-Flight Secondary Ion Mass Spectrometry (ToF-SIMS)

A ToF-SIMS M6 instrument (IONTOF, Münster, Germany) was used to perform depth profiling and corresponding 3D imaging of PEF-based nanocomposites. A primary ion beam Bi_3_^+^ operated at a target current of 0.6 pA. The analysis area was 300 × 300 µm. Sputtering was performed with a 5 keV gas cluster ion beam (GCIB) rastering over 500 × 500 µm. During the measurements, the flood gun was on, and a surface potential of –500 V, Ar gas flooding (5·10^–7^ torr), and topography mode was applied. Mass calibration was performed using the peak at known mass-to-charge (*m*/*z*) ratio: a peak at *m*/*z* 12.00 for C^+^, a peak at *m*/*z* 27.02 for C_2_H_5_^+^, and a peak at *m*/*z* 41.04 for C_3_H_5_^+^.

#### 2.3.5. Scanning Electron Microscopy (SEM)

SEM analysis of the nanocomposites was carried out using a JEOL (Tokyo, Japan) 2011 (JMS-840) scanning microscope at 10–20 kV equipped with an energy-dispersive X-ray spectroscopy detector an Oxford (Abingdon, UK) ISIS 300 microanalytical system. In the case of sample preparation, all surfaces were coated with carbon black to avoid charging under the electron beam.

#### 2.3.6. Differential Scanning Calorimetry (DSC)

The thermal transitions were studied by conventional calorimetry employing a TA Q200 DSC instrument (TA Instruments, New Castle, DE, USA), calibrated with sapphires for heat capacity and indium for temperature and enthalpy. Pieces of the prepared samples of ~6–7 mg in mass were closed in aluminum Tzero TA pans and studied within the temperature range 0 to 260 °C in a high-purity nitrogen atmosphere (99.9995%). The polymers were heated up to 260 °C to erase any thermal history and achieve the best thermal contact between the sample and the aluminum pan. Subsequently, two cooling–heating runs were performed, i.e., one involving a fast cooling (scan 1, 100–110 °C/min, in the region of expected crystallization) and another involving a conventional cooling rate (scan 2, 20 °C/min), to produce amorphous and semicrystalline polymers, respectively (Figure S5). Finally, the subsequent heating scans were recorded at a 10 °C/min fixed heating rate.

A PerkinElmer Pyris DSC-6 differential scanning calorimeter, calibrated with pure indium and zinc standards, was used to determine the thermal transitions of the as-received materials. Samples of 5 ± 0.1 mg were sealed in aluminum pans to measure the thermal characteristics of annealed samples. All experiments were performed under an N_2_ atmosphere with a 20 mL/min flow. Crystallinity degree (*X_c_*) was calculated with Equation (3):

(3) $$X_{c}\;(\%)=\left(\frac{{\Delta H}_{m}-{\Delta H}_{cc}}{(1-w)\times {\Delta H}_{f}^{0}}\right)\times 100$$

where ${\Delta H}_{m}^{0}$ = 137 J/g, as reported by our team in our previous work for the heat of melting of the 100% crystalline PEF [20].

#### 2.3.7. X-ray Diffraction (XRD)

XRD was employed to study the semicrystalline structure of all samples at RT that had previously been melted and subsequently annealed (160 °C, 1 h). The XRD spectra were recorded using a MiniFlex II XRD system (Rigaku Co., Tokyo, Japan), with Cu Ka radiation (0.154 nm), over the 2θ range from 5° to 50° with a scanning rate of 1°/min. The percentage crystallinity was calculated from the XRD graphs using Equation (4):

(4) $$X_{c}={\left(1+\frac{A_{am}}{A_{c}}\right)}^{-1}$$

*A_am_* is the area of the amorphous halo, and *A_c_* is the area of the crystalline peaks.

#### 2.3.8. Broadband Dielectric Spectroscopy (BDS)

The molecular dynamics, emphasizing segmental mobility, were studied employing BDS in a nitrogen atmosphere [21]. The recordings were made by a Novocontrol BDS setup, namely, an alpha frequency response analyzer (FRA) combined with a Novocontrol Quatro liquid nitrogen cryosystem (Novocontrol GmbH, Montabaur, Germany). The measurements were performed on initially amorphous samples (melted and fast-cooled) using a sandwich-like capacitor of 20 mm in diameter and ~1.2 mm in the electrode distance (sample thickness). The complex dielectric permittivity, ε* = ε′ − i·ε″, was recorded isothermally as a function of frequency in the range from 10^−1^ to 10^6^ Hz and in the temperature range from −120 up to 150 °C on heating at steps of 5 and 10 K, depending on the process followed.

#### 2.3.9. CA Measurements

CA measurements of all the PEF samples were taken using a goniometer from DataPhysics (Filderstadt, Germany) and were performed with ultrapure water (Millipore, Burlington, MA, USA) with a droplet volume of 3 μL. An average value with standard deviation was calculated. CA of the samples was measured at room temperature. The statistical evaluation was conducted through a one-way ANOVA followed by a post hoc Tukey test, facilitated by the GraphPad Prism 6 software. A *p*-value of less than 0.05 was deemed as indicative of statistical significance.

#### 2.3.10. Antibacterial Properties

The antimicrobial activity of neat PEF and its nanocomposite films against the bacteria *Escherichia coli* (DSM 1576) and *Staphylococcus aureus* (DSM 799) was determined according to the internal protocols of the Department of Microbiological Research, Center for Medical Microbiology of the National Laboratory for Health, Environment, and Food in Maribor, i.e., No. P96 Biofilm production on various materials P90 (ISO22196)—for determination of microbiological activity/antimicrobial activity of the samples’ surfaces. More specifically, films of 10 mm × 10 mm were exposed to the standardized medium inoculated with *Escherichia coli* and *Staphylococcus aureus* and set at 0.5 on the McFarland scale. The antimicrobial performance of the neat PEF and PEF nanocomposite films was determined after 6 h. After injecting the neat PEF and PEF nanocomposite films, the viable bacteria were evaluated by the pour-plate method (plate counting agar was used). The effect of incorporation of NPs into the PEF matrix was evaluated as a reduction in bacterial growth and counting of bacterial number after incubation of neat PEF compared to PEF nanocomposite films. The neat PEF and nanocomposite films’ antibacterial effectiveness against *S. aureus* and *E. coli* is reported as the mean standard deviation (SD) after 6 h of contact. Each experimental procedure was replicated three times (n = 3) for each bacterium strain. For statistical analysis, two-way ANOVA with repeated measurements was performed.

#### 2.3.11. Nanoindentation Tests

Nanoindentation was used to investigate the mechanical performance of neat PEF and PEF nanocomposite materials. Nanoindentation testing was carried out using a dynamic ultra-microhardness tester DUH-211 (Shimadzu Co., Kyoto, Japan) with a 100 nm radius triangular pyramid indenter tip (Berkovich-type indenter) at room temperature (296 K). Using a diamond tip, a controlled load (P) with a peak load at 200 mN (held for 3 s) was applied to the film’s surface. The nanoindentation depth was determined as a function of load. Then, the indenter was unloaded, leading to a load of zero. During the creep time, the maximum load is applied to the intender. The average value of 10 measurements was used to calculate the elastic modulus and hardness of neat PEF and their PEF nanocomposite specimens. Data analysis was performed using one-way ANOVA, followed by a post hoc Tukey test, facilitated by GraphPad Prism 6 software. A *p*-value under 0.05 was established as the threshold for statistical significance.

## 3. Results

### 3.1. Synthesis of PEF-Based Nanocomposites

The PEF was synthesized via a two-stage melt polycondensation technique using a Sb_2_O_3_ catalyst, resulting in [*ղ*] of 0.43 dL/g. The synthesized PEF-based nanocomposites exhibited [*ղ*] values ranging from 0.38 dL/g to 0.48 dL/g, as displayed in Table 1. As observed, there is no significant increase in [*η*] values after incorporating the active agents into the polymer matrix. Furthermore, PEF–ZnO nanocomposite materials exhibit decreased [ղ] value compared to the neat PEF, indicating that incorporating ZnO nanoparticles affects the polymerization reaction process [21]. Among the nanocomposites, the PEF–bioglass sample displayed the highest [*ղ*] value (0.48 dL/g). The structural properties of Ce–bioglass nanoparticles are detailed in Table S1 and Figures S2–S5. Thus, it can be concluded that the kind of inorganic additives used slightly affects the molecular weight value of the final material [22]. The number average molecular weight (*M_n_*) values of the synthesized composites are presented in detail in Table 1.

**Figure 1:**
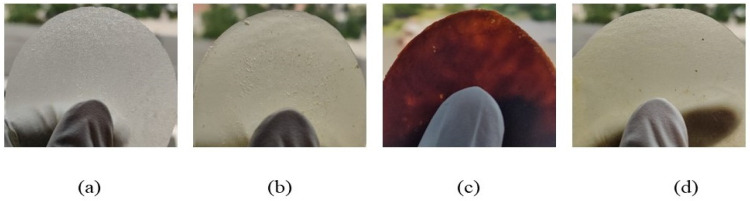
The appearance of (**a**) neat PEF, (**b**) PEF–bioglass, (**c**) PEF–ZnO, and (**d**) PEF–ZrO_2_ composite films.

The appearance of the neat PEF and PEF-based nanocomposites is illustrated in Figure 1. As seen, neat PEF is bright white due to the monomer’s high purity and the use of Sb_2_O_3_ as a catalyst [23] (Figure 1a). Furthermore, all the PEF-based nanocomposites showed an increase in color concentration compared to the neat PEF sample. The colorimetric data of all the synthesized polymers are displayed in Table 2.

Among the nanocomposites, PEF–bioglass exhibited higher L*, h° values, and lower K/S values, indicating the lower color concentration of PEF–bioglass film, as illustrated in Figure 1b. On the contrary, the PEF–ZnO sample showed significantly lower L* and h° values and higher a* and K/S values (Figure 1c), making it suitable for light-sensitive food packaging that requires opaque coatings [24]. Based on these results, it can be concluded that PEF–bioglass and PEF–ZrO_2_ have similar transparency and can be used for food packaging applications.

### 3.2. ATR-FTIR Spectroscopy

**Figure 2:**
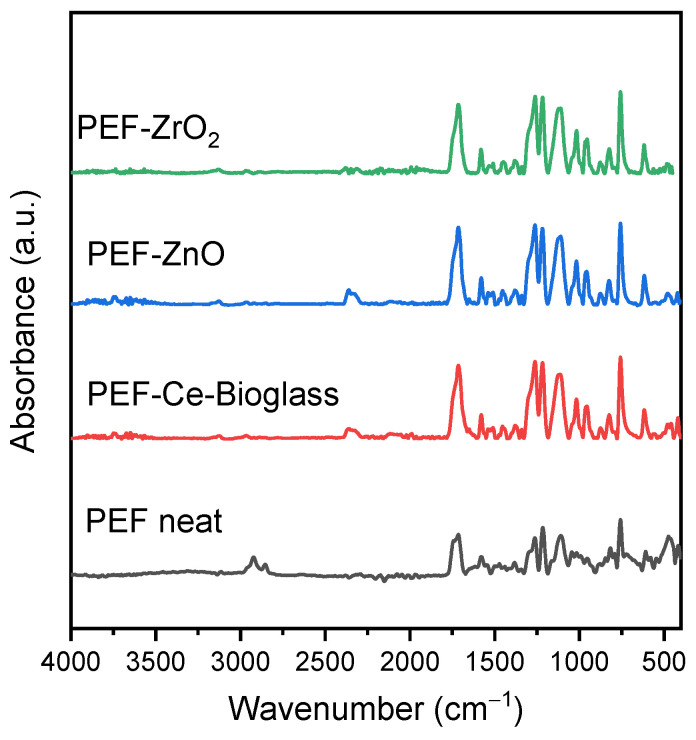
ATR-FTIR spectra of the neat PEF and PEF-based nanocomposite samples.

The ATR-FTIR spectra of PEF and PEF-based nanocomposite samples are presented in Figure 2. The measured ATR-FTIR spectrum of all the samples show peaks as follows: 3126–3163 cm^−1^ (C-H stretching vibrations of the furan ring); 2924–2933, 2853–2872 cm^−1^ (asymmetric and symmetric C-H stretching vibrations); 1713–1727 cm^−1^ (C-O stretching vibrations); 1578–1583 cm^−1^ (aromatic C-C bending vibrations); 1508–1512, 1453–1458 cm^−1^ (C-H deformation and wagging vibrations); 1374–1386 cm^−1^ (C-H rocking vibrations); 1262–1264, 1222–1229 cm^−1^ (C-O stretching vibrations); 1107–1113, 1011–1022 cm^−1^ (C–O–C) ring vibrations, furan ring); 944–953, 813–823, and 760–765 cm^−1^ (C-H out-of-plane deformation vibrations, furan ring); 612–620 cm^−1^ (C-H bending vibrations). As observed, the characteristic peaks of PEF appeared in all spectra, revealing successful polymerization in all cases [25]. Among the nanocomposites, PEF–bioglass and PEF–ZnO samples showed a low-intensity absorption peak in the range of 3654–3668 cm^−1^, corresponding to the O-H stretching vibrations of the hydroxylic group of the nanoparticles, indicating the effective incorporation of the additives into the polymeric matrix [26].

### 3.3. ToF-SIMS Depth Profiling of the Nanocomposite Samples

The 3D depth profiling of nanocomposites was performed using a GCIB sputtering source so as not to change the chemistry of the analyzed sample during the sputtering procedure [27,28].

**Figure 3:**
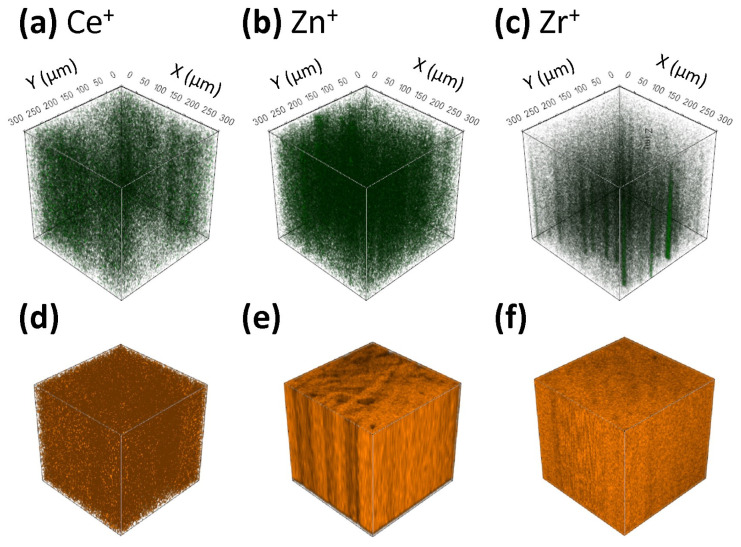
ToF-SIMS 3D images showing the spatial distribution of signals for (**a**) Ce^+^ in PEF–bioglass nanocomposite, (**b**) Zn^+^ in PEF-ZnO nanocomposite, and (**c**) Zr^+^ in PEF-ZrO_2_ nanocomposite. The distribution of the C_4_H_3_^+^ signal representing the polymer matrix for (**d**) PEF-bioglass nanocomposite, (**e**) PEF-ZnO nanocomposite, and (**f**) PEF-ZrO_2_ nanocomposite. Darker (black) regions in the 3D images represent lower concentrations of the corresponding species.

Figure 3 shows the distribution of the Ce^+^, Zn^+^, and Zr^+^ signals originating from CeO_2_ in PEF-bioglass, ZnO in PEF-ZnO nanocomposite, and ZrO_2_ in PEF-ZrO_2_ nanocomposite. The distribution of the Ce^+^ signal in PEF–bioglass was analyzed because of the antimicrobial properties of CeO_2_. Even though a low amount of CeO_2_ (1 wt.%) was incorporated in the nanoparticles, the signal for Ce^+^ was intense using the ToF-SIMS technique. Figure 3a shows that Ce^+^ is homogeneously distributed in the PEF-bioglass.

The distribution of Zn^+^ originating from ZnO in the PEF-ZnO nanocomposite is shown in Figure 3b. ZnO is the active agent incorporated into the PEF matrix to improve the antimicrobial properties of the composite. The 3D ToF-SIMS image shows that the topmost position on the surface contains a lower concentration of ZnO. On the other hand, by sputtering deeper into the subsurface region, the signal for Zn^+^ becomes more intense, indicating that the ZnO content is higher in the bulk material, where it is homogeneously distributed.

ZrO_2_ is the active agent incorporated into the PEF matrix. Similarly to the Zn^+^ signal in the PEF-ZnO nanocomposite, the signal for Zr^+^ originating from ZrO_2_ increases by sputtering deeper into the subsurface region of the PEF-ZrO_2_ nanocomposite sample (Figure 3c). Therefore, the amount of ZrO_2_ is low on the topmost position of the surface, whereas its abundance increases in the bulk material.

Figure 3d–f show the homogeneous distribution of the C_4_H_3_^+^ signal for Figure 3d PEF-bioglass nanocomposite, Figure 3e PEF-ZnO nanocomposite, and Figure 3f PEF-ZrO_2_ nanocomposite.

### 3.4. SEM-EDX

**Figure 4:**
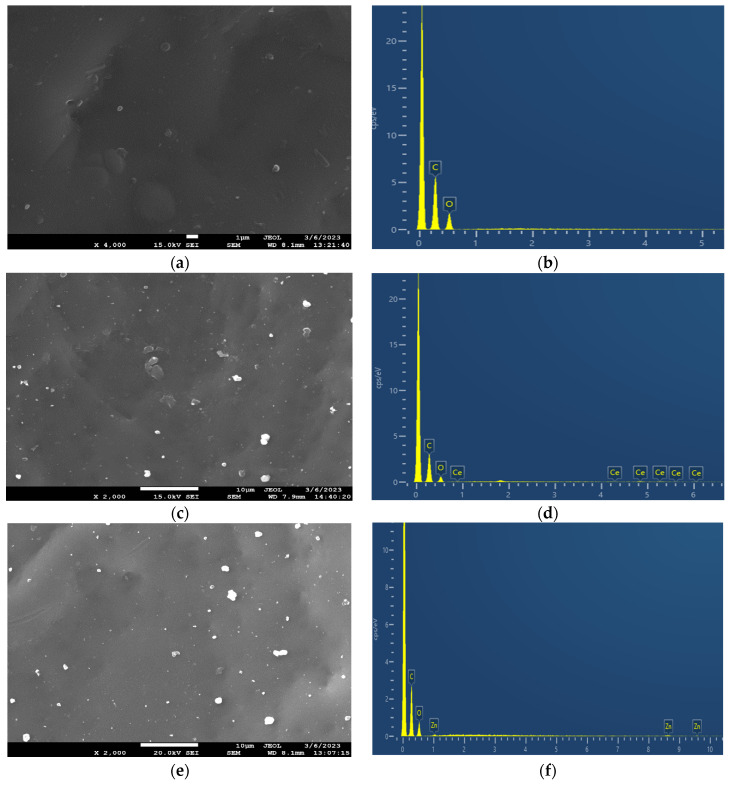

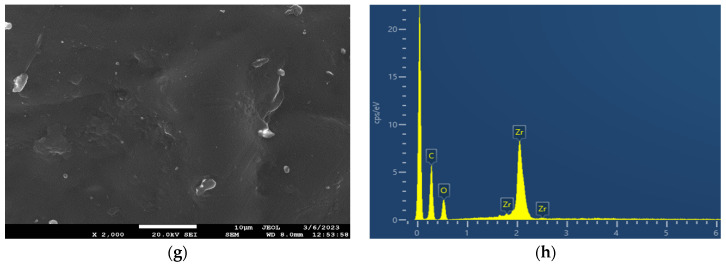
SEM images of (**a**) neat PEF and (**c**) PEF–bioglass, (**e**) PEF–ZnO, and (**g**) PEF–ZrO_2_ composites. EDX spectra of (**b**) neat PEF and (**d**) PEF–bioglass, (**f**) PEF–ZnO, and (**h**) PEF–ZrO_2_ composite materials. The SEM photograph of neat PEF was taken using magnification ×4000, while the ones of the composites were taken in ×2000 magnification mode.

The surface morphology and the elemental composition of the neat PEF and PEF-based nanocomposites were investigated through SEM-EDX. The SEM micrographs of all samples are displayed in Figure 4. The neat PEF sample exhibited a smooth surface without cracks, as observed in Figure 4a. The incorporation of active agents into the PEF matrix provoked small aggregates spread across the surface of the matrix, as illustrated in Figure 4c,e,g. The low concentration (1 wt.%) of nanoparticles hinders the formation of clusters and the agglomeration of particles in the polymer matrix. Based on the literature, it is evident that an increase in the concentration of nanoparticles leads to clusters and agglomeration of particles in the polymer matrix, affecting nanocomposites’ properties. Furthermore, the in situ polymerization technique prevents the agglomeration of particles through the direct integration of well-dispersed active agents within the PEF matrix [29].

The EDX spectra of neat PEF and PEF-based nanocomposites exhibited two peaks. The first strong peak at 0.2 keV is related to the carbon atom, and the second small peak at 0.5 keV is related to the oxygen atom of PEF polyester. The presence of Ce-, Zn-, and Zr-related peaks in Figure 4d,f,h indicates the successful incorporation of the nanoparticles in the PEF matrix.

### 3.5. Thermal Properties and Crystallinity

**Figure 5:**
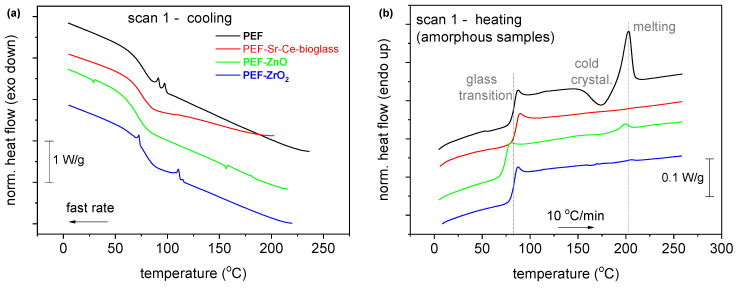

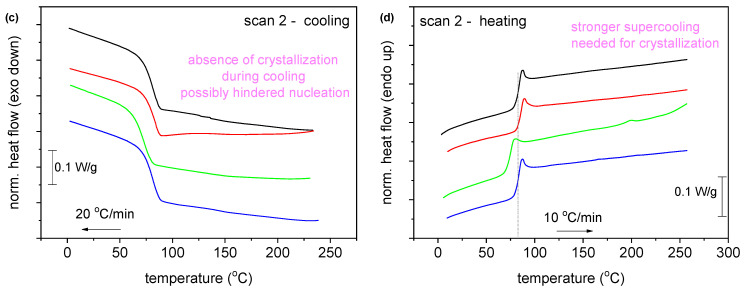
Comparative DSC traces of the PEF-based nanocomposites and neat PEF for both thermal scans (**a**) during quenching, (**b**) subsequent heating after quenching, (**c**) cooling with 20 °C/min and (**d**) subsequent heating after (**c**). The shown heat flow has been normalized to each sample mass. The vertical lines have been added to mark effects on some thermal events recorded in the nanocomposites compared to neat PEF.

The thermal analysis of the synthesized polymeric materials is demonstrated in Figure 5. As can be observed, the glass transition temperature *T*_g_, (in the amorphous samples) exhibits a direct dependence on the low *M*_n_s, as for the lower *M*_n_ (7.4 k), the lower *T*_g_ (74 °C) is recorded (Figure 5, Table 3). Due to the low *M*_n_, nucleation and crystal growth are quite hindered. Thus, the crystalline fractions, CF, are estimated to be between 0% and 5%. Furthermore, neat PEF exhibits a clear ability to crystallize. Considering that *M_n_* can both increase and decrease in the nanocomposites, this result could indirectly indicate the strong interfacial polymer/filler interactions. The latter exists ab initio due to the in situ polymer nanocomposite synthesis. Interestingly, upon fast cooling, neat PEF and PEF–ZrO_2_ exhibit weak cold crystallization and melting during the subsequent heating. The results suggest that strong supercooling is necessary for nucleation, an effect that is expected due to short polymer chains along with the rigid backbone originating from the existence of the large rigid furan ring [30].

**Figure 6:**
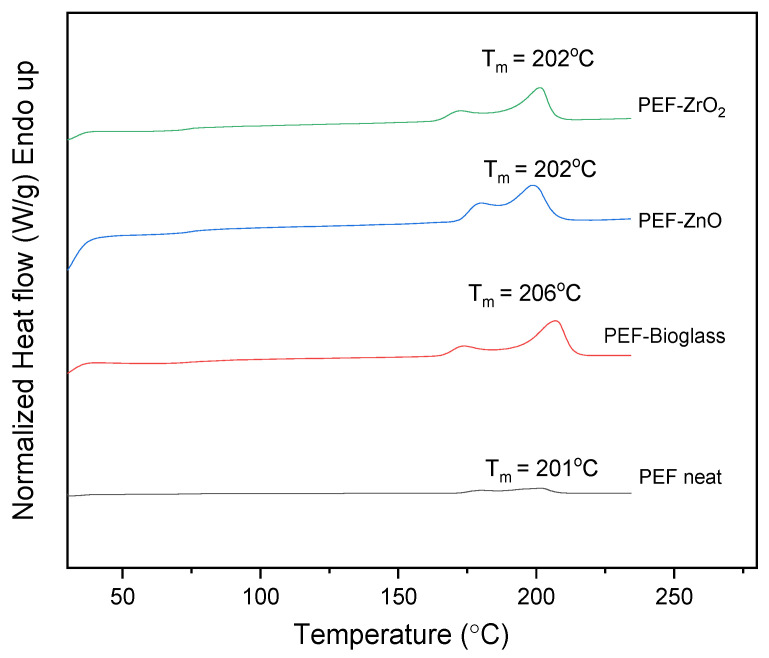
DSC scans of neat PEF and PEF-based nanocomposites after annealing (1st heat, rate 20 °C/min).

The DSC scans of all samples, after annealing for 1 h at 160 °C with a heating rate of 20 °C/min, are presented in Figure 6. Specifically, two melting peaks, *T_m_*_1_ and *T_m_*_2_, are observed in all cases. The second melting peak, *T_m_*_2_, at higher temperatures is due to the melting–recrystallization–melting process, a common behavior of furan-based polyesters [31,32,33]. The neat PEF sample showed an increase in the degree of crystallinity (*X_c_*) after annealing, as reported in Table 4. The *X_c_* of PEF-based nanocomposites was increased by more than 20%, indicating that incorporating nanoparticles into the PEF matrix did not affect the recrystallization rate.

**Figure 7:**
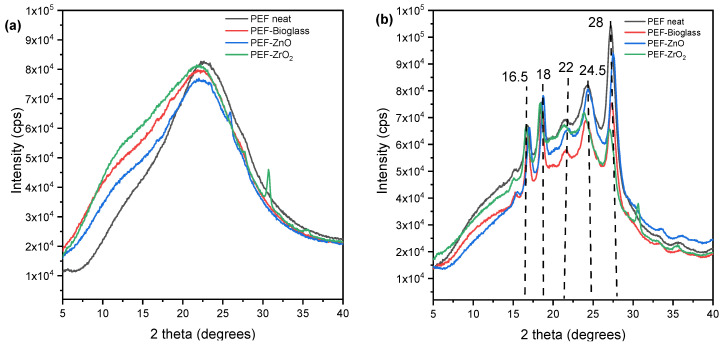
XRD patterns of (**a**) neat PEF and PEF based nanocomposites (amorphous), (**b**) neat PEF and PEF-based nanocomposite (annealed). Annealing is done at 160 °C for 1 h.

The XRD patterns of neat PEF and PEF-based nanocomposites are displayed in Figure 7. Figure 7b illustrates the XRD patterns of the annealed neat PEF and PEF-based nanocomposites. The main characteristic diffraction peaks of PEF were observed at 2θ ≈ 16.5°, 2θ ≈ 18°, 2θ ≈ 22°, 2θ ≈ 24.5°, and 2θ ≈ 28° in all cases. Furthermore, the *X_c_* was calculated from A_am_ and A_c_ using equation (4) through origin software and was displayed in Table 4. As observed, the annealing process increased the degree of crystallinity of the samples. Hence, it is understood that the incorporation of nanoparticles into the PEF matrix did not affect the improvement in crystallinity after annealing.

### 3.6. Broadband Dielectric Spectroscopy

BDS enables the evaluation of molecular mobility via the dipolar relaxation motions of local polymer segments (*β* relaxation here) and the overall polymer chain’s segmental motions (*a* relaxation here). Actually, *a* relaxation is considered the dielectric analogue of the calorimetric glass transition.

**Figure 8:**
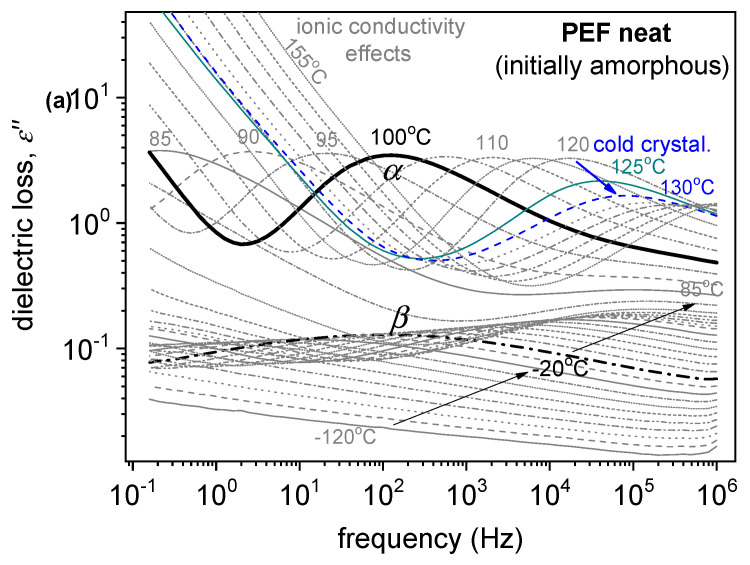

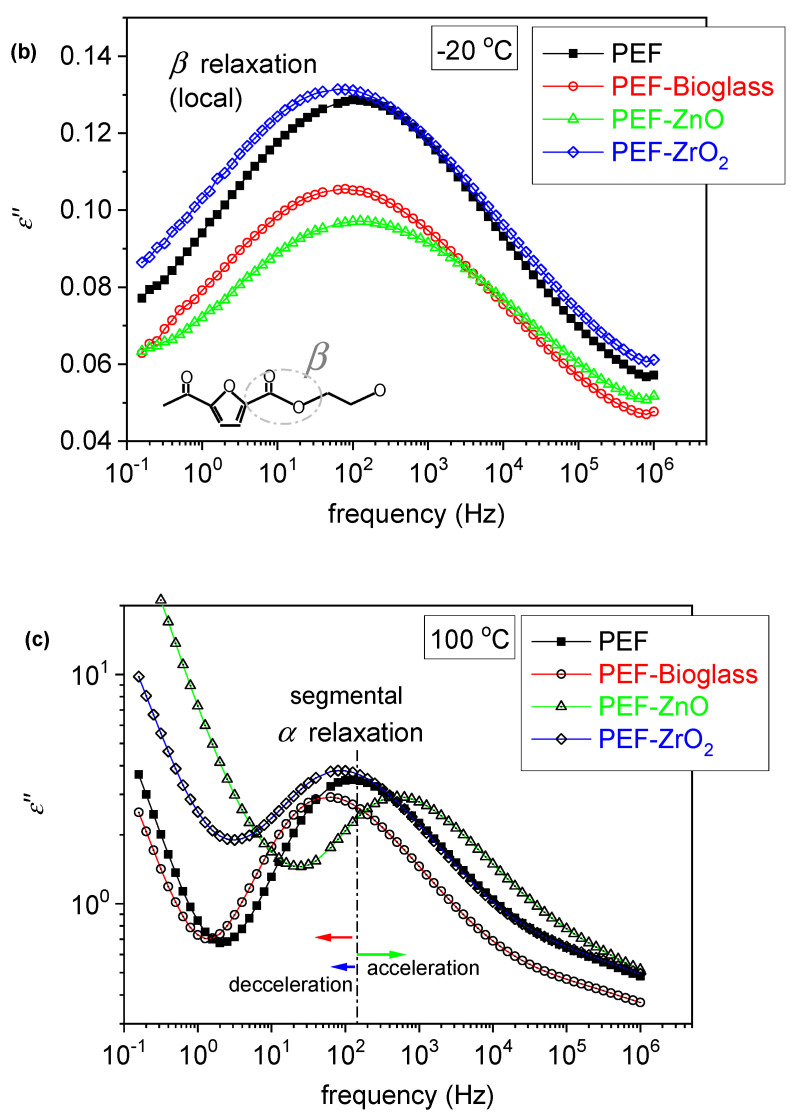
(**a**) BDS raw data of neat amorphous PEF in the form of the imaginary part of dielectric permittivity, *ε*″, against frequency, shown for various temperatures. (**b**,**c**) Comparative isothermal *ε*″(*f*) results for all samples at two selected temperatures, (**b**) one below *T*_g_ showing the local *β* process and (**c**) one above *T*_g_ to present the effects imposed on the segmental *α* process.

The dielectric relaxation processes are followed here, as in many studies in the literature, as peaks in the frequency, *f*, dependents of the imaginary part of dielectric permittivity, *ε*″(*f*). Examples are presented in Figure 8. As expected, when temperature, *T*, increases, any mobility is accelerated. Thus, the corresponding *ε*″(*f*) peak migrates toward higher frequencies. This proves that BDS indirectly follows the polymer molecular dynamics.

**Figure 9:**
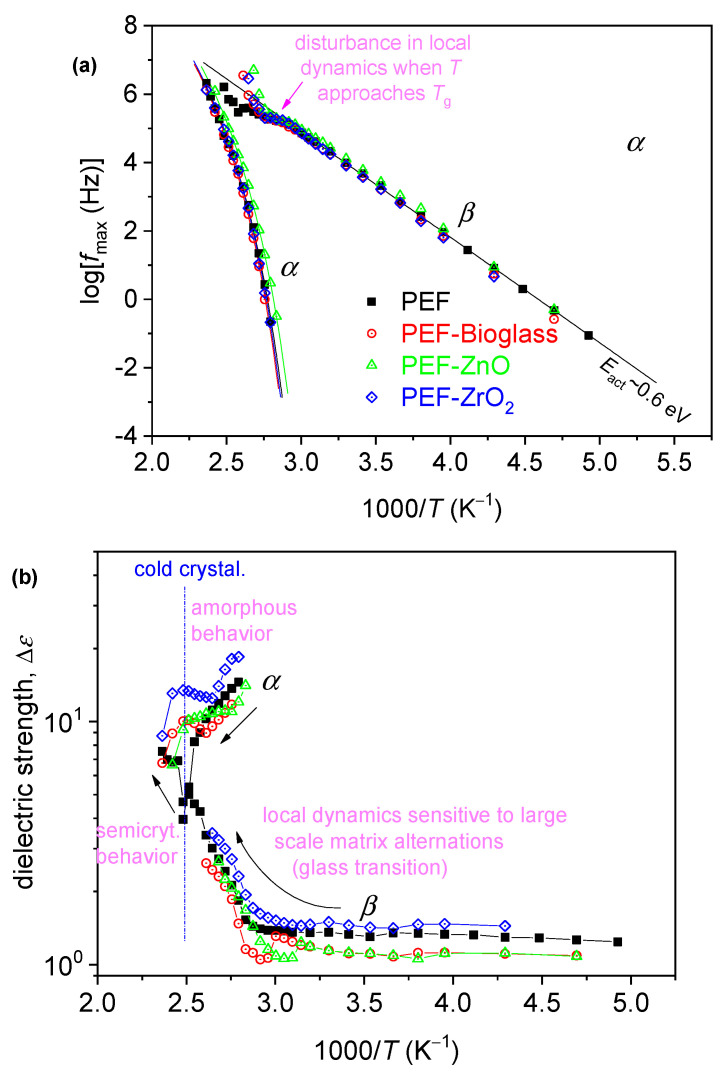
The dielectric activation map for neat PEF and the PEF-based nanocomposites, in terms of the reciprocal temperature dependence of (**a**) frequency maxima of the recorded relaxation peaks, log*f*_max_, and (**b**) the corresponding dielectric strengths, Δ*ε*. In (**a**), the added straight and curved lines connecting the experimental points are fittings of Arrhenius and the Vogel–Tammann–Fulcher–Hesse equations [34].

Our results have been analyzed and evaluated by widely adopted routes [34,35], involving the fitting of *ε*″(*f, T*) by model mathematical functions (not shown) and construction of the dielectric activation map in terms of timescale and dielectric strength, Δ*ε* (Figure 9). Then, the timescale data of Figure 9a were also fitted by Arrhenius and Vogel–Tammann–Fulcher–Hesse equations to estimate the activation energy, *E*_act_, of the local relaxation, and the fragility, *m*_α_, and dielectric *T*_g_ (*T*_g, diel_) of *α* relaxation [36,37], respectively. The latter two values have been included in Table 5.

The recording of local *β* and segmental *α* dynamics is shown in Figure 9. The segmental *α* relaxation is recorded as a peak in the *ε*″(*f*) spectra at *T* closely above *T*_g_; thus, it is the “dielectric analogue of the glass transition.” The local relaxation exhibits a similar timescale in all samples, whereas the segmental one follows qualitatively the effects of *T*_g_, namely, slight deceleration in the PEF–bioglass and PEF–ZrO_2_, but mild acceleration in PEF–ZnO (Figure 9). Furthermore, the values for the estimated dielectric *T*_g_, T_g, diel_ are displayed in Table 5. As reported in the calorimetric data, the alternations are compatible with the changes in *M*_n_, in qualitative agreement with the DSC data. There are also no significant alternations in the chain’s fragility/cooperativity with *m*_α_~100 (Table 5). This is expected due to the short polymer chains (low *M*_n_). In general, the rigid backbone of poly(*n*-alkylene furanoate). The dielectric strength, Δ*ε*, and dependence from *T* are as expected for both *β* and *α*, and in relation to the initially amorphous state and the evolution of cold crystallization at elevated temperatures [38].

### 3.7. CA Analysis

**Figure 10:**
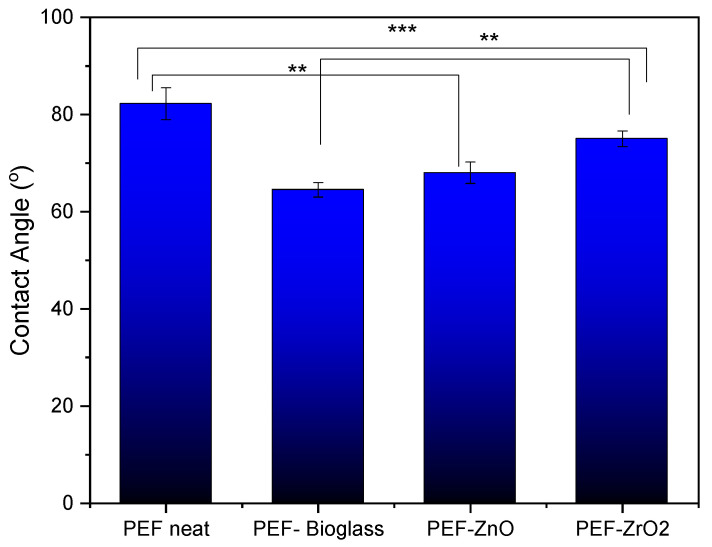
Average CA measurements with standard deviation of neat PEF and PEF nanocomposite films. One-way ANOVA. ** 0.001 < *p* < 0.01, *** 0.0001 < *p* < 0.001.

In order to determine the hydrophobicity of the synthesized materials, water CA measurements were performed. Figure 10 shows the water CA values of neat PEF and PEF nanocomposite films, measured at room temperature. The average CA value of all PEF and PEF nanocomposite films was less than 90°, indicating its hydrophilic nature. Incorporating active agents into the PEF matrix significantly reduces the CA due to the formation of aggregates that increase surface roughness, as observed in SEM images (Figure 4). Phothisarattana et al. synthesized polybutylene adipate-co-terephthalate (PBAT) and thermoplastic starch (TPS) blended ZnO (1–5%) nanocomposite films. The author reported that increasing amounts of ZnO increased the size of the dispersed particles and due to aggregates of nanoparticles causes reduction in hydrophobicity of films [39]. In a similar work, Zhu et al. synthesized PEF nanocomposite films by incorporating ZnO nanoparticles (ZnO NPs) through solvent casting and observed that the composite film’s CA values decreased compared to the neat one ascribed to the increased surface roughness [13].

The relationship between surface roughness and CA is determined not only by the roughness itself but also by the chemical properties of the surface, the properties of the liquid (e.g., surface tension), and the presence of polar or nonpolar functional groups. It should be noted that as metals are incorporated into the surface, their surface free energy increases enormously, and thus wetting with water with a surface tension of 73 mN/m is very good [40]. The hydroxyl groups are very polar and thus increase the hydrophilicity of the materials and allow good wetting with water. PEF–bioglass and PEF–ZnO nanocomposite films exhibit a contact angle of less than 70°, attributed to the presence of hydrophilic hydroxyl groups, as reported in ATR-FTIR spectroscopy measurements. The hydroxyls can form hydrogen bonds with water molecules, which causes stronger interaction between water molecules and the film’s surface, lowering the CA value [41].

### 3.8. Antimicrobial Studies

**Figure 11:**
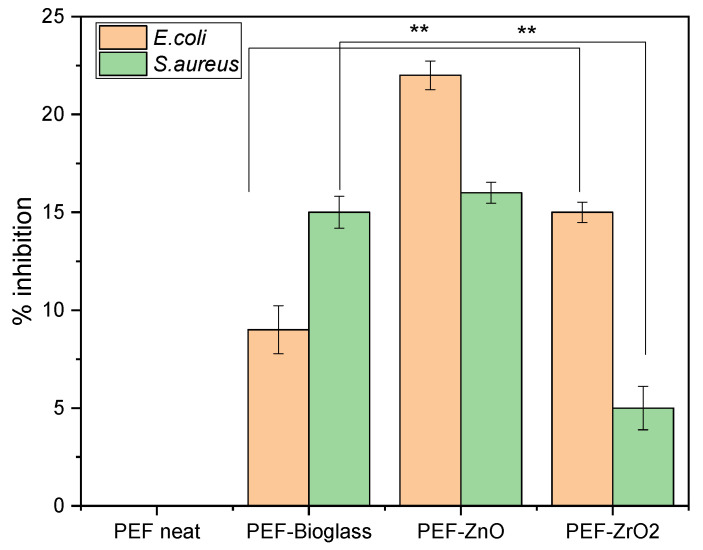
Antimicrobial activity of neat PEF and PEF nanocomposite films against *E. coli* and *S. aureus*. Two-way ANOVA with repeated measurements, ** 0.001 < *p* < 0.01.

The antimicrobial activity of *E. coli* and *S. aureus* as representatives of Gram-negative and Gram-positive bacterial strains, respectively, on neat PEF films and PEF nanocomposite films after 6 h incubation time is summarized in Figure 11. An increase in percentage inhibition was observed against both the bacterial strains in all the nanocomposite samples in relation to the neat PEF. Based on the literature, the increase in the percentage weight fraction of the active agents incorporated into the polymer matrix has not shown increased antimicrobial activity. Instead, it leads to clusters and agglomeration of particles, which also increases the surface roughness of the composites [42]. The results show that the percentage inhibition of nanocomposite films is better than neat PEF, but still not so great as to be antimicrobial-active. However, the increased inhibition rate against *E. coli* and *S. aureus*, higher concentrations of these metals, and their availability on the surface show improved bacterial inhibition. As observed in the ToF-SIMS report, only a few nanoparticles were observed on the surface of the films due to the incorporation of a low fraction by weight of nanoparticles (1 wt.%) into the PEF matrix.

Raghupathi et al. investigated the impact of ZnO particle size on antibacterial activity. The results indicated that the inhibition of *S. aureus* was incomplete when the particle size was greater than 100 nm. Additionally, confocal microscopy showed that treating bacteria with small nanoparticles led to increased cell death due to the rupture of the bacterial cell wall [42]. Promhuad et al. reported that surface interactions between positively charged ZnO nanoparticles and negatively charged bacteria developed reactive oxygen species and results in cell death of bacteria [43]. In the present work, PEF–ZnO nanocomposite films exhibit more than 15% inhibition of *E. coli* and *S. aureus* due to the selection of ZnO NPs less than 100 nm.

PEF–bioglass and PEF–ZrO_2_ nanocomposite films showed less than 15% inhibition of *E. coli* and *S. aureus,* but increased antimicrobial activity was recorded compared to a neat PEF sample. Farias et al. reported that, e.g., Ce-based nanoparticles showed antimicrobial activity against Gram-positive and Gram-negative bacteria but with more significant antimicrobial activity against Gram-negative bacteria like *E. coli.* Gram-positive bacteria have a thick layer of peptidoglycan that contains linear polysaccharide chains with short peptides forming a rigid structure and making Ce-based nanoparticles difficult to penetrate [44,45]. Similarly, PEF–bioglass nanocomposite film shows higher antimicrobial activity against Gram-negative *E. coli*, as seen in Figure 11.

Thamir et al. studied the antimicrobial activity of ZrO_2_NPs against *E. coli* and *S. aureus.* They found that ZrO_2_NPs led to reactive oxygen species (ROS) formation, which destroyed bacterial cells, such as lipid peroxidation. As a result, the destruction of bacterial cells leads to bacteria death and increases the substrate’s microbial resistance. Also, metal oxide nanoparticles induce oxidation in microorganisms, which results in their death. In our present work, synthesized PEF–ZrO_2_ nanocomposite films showed improved antimicrobial activity against *E. coli* and *S. aureus* [46].

### 3.9. Nanoindentation

**Figure 12:**
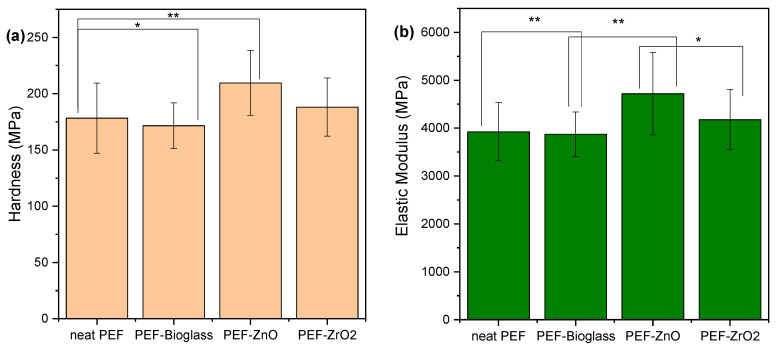
Effect of different nanoparticles on the (**a**) hardness and (**b**) elastic modulus of PEF matrix composites. One-way ANOVA. * *p* 0.01–0.05, ** 0.001 < *p* < 0.01.

The findings from tests conducted on PEF specimens reinforced with various nanoparticles reveal intriguing patterns in the nanoindentation investigation of these composite materials (as shown in Figure 12). The hardness of neat PEF was measured at 178.29 MPa, and this value increased in the presence of nanoparticles, reaching 209.55 MPa for ZnO and 188.04 MPa for ZrO_2_. Conversely, in the case of the bioglass nanocomposite, a slight decrease resulted in a hardness of 171.68 MPa. A similar trend was observed in elastic modulus measurements, with neat PEF exhibiting 3925 MPa, while ZnO, ZrO_2_, and the Ce–bioglass yielded values of 4719 MPa, 4176 MPa, and 3873 MPa, respectively. It is worth noting that the enhanced impact of ZnO on mechanical properties has been previously reported in PLA-reinforced composites by Črešnar et al. [40]. However, these comparisons were made against TiO_2_ and Ag nanoparticles. Finally, the increased values of the ZnO nanocomposite sample can be attributed to the higher *X_c_* that this sample exhibits in combination with its smoother surface.

## 4. Conclusions

PEF-based nanocomposites incorporating different active agents such as nanoparticles of ZnO and ZrO_2_ and a Ce–bioglass were synthesized via an in situ polymerization technique. The intrinsic viscosity and molecular weight values of the PEF-based nanocomposites were determined. The PEF–ZnO sample showed increased color concentration with high K/S, a*, and low L* values in color measurements. Among the PEF-based nanocomposites, PEF–bioglass had the lowest color concentration with low K/S and high L* values. PEF–bioglass and PEF–ZnO nanocomposites exhibited small O-H stretching vibrations analyzed with ATR-FTIR spectroscopy. The elemental composition and spatial distribution of active agents in the polymer matrix were determined using ToF-SIMS depth profiling. SEM images show that due to the low concentration (1 wt.%) of nanoparticles, clusters and agglomeration of particles in the polymer matrix are avoided. The DSC traces showed that the neat PEF and PEF nanocomposite samples showed CF between 0% and 5%. A weak cold crystallization and melting of ZnO nanocomposite is observed during heating. The annealed neat PEF and PEF nanocomposite samples showed an increase in crystallinity above 20%. BDS results did not reveal significant or systematic effects in *Δε* for the polymer nanocomposites. Furthermore, the contact angle values of nanocomposites decrease due to an increase in the surface roughness of nanocomposites and polar group’s introduction. Concerning the antimicrobial studies, the PEF-based nanocomposites with all active agents showed increased inhibition of 5–16% for Gram-negative bacterial strains and 9–22% for Gram-positive bacteria strains. The nanoindentation results show that the PEF–ZnO nanocomposite displayed enhanced hardness and elastic modulus compared to neat PEF. A small increase has also appeared in PEF–ZrO_2_, while PEF–bioglass has almost similar hardness and elastic modulus values. Based on these results, it could be concluded that PEF–ZrO_2_ and PEF–bioglass could be appropriate for food packaging applications thanks to their low coloration and antimicrobial potential. The Ce–bioglass developed in this study can be used as a reinforcement agent to improve the mechanical and antimicrobial properties of food packaging films and containers.

## Figures and Tables

**Table 1:** Values of [*ղ*] and number average molecular weight values of prepared nanocomposites.

Sample	Active Agents	[*ղ*] (dL/g)	*M_n_* (g/mol)
PEF neat	-	0.43	8900
PEF–bioglass	Ce–bioglass (78.5SiO_2_-10SrO-10P_2_O_5_-0.5MgO-1CeO_2_)	0.48	10,600
PEF–ZnO	ZnO	0.38	7400
PEF–ZrO_2_	ZrO_2_	0.45	9600

**Table 2:** Colorimetric data L*, a*, b*, c*, h°, and K/S values of the neat PEF and PEF-based nanocomposite samples.

Sample	L*	a*	b*	c*	h°	R	K/S
PEF neat	90.07	−2.21	7.49	7.47	107.57	47.14	0.3
						(400 nm)	
PEF–bioglass	78.27	−0.78	26	25.74	91.84	15.87	2.2
						(400 nm)	
PEF–ZnO	27.85	12.77	1.90	3.94	32.26	4.93	9.1
						(480 nm)	
PEF–ZrO_2_	76.06	0.08	26.55	26.45	90.38	14.63	2.5
						(400 nm)	

**Table 3:** Measured values of the materials under investigation for the molar mass, *M*_n_, and values obtained by DSC: glass transition temperature, *T*_g_, heat capacity change during glass transition, Δ*c*_p_, crystallization and cold crystallization temperatures, *T*_c_ and *T*_cc_, respectively, a crystalline fraction (CF)* estimated by the enthalpies of crystallization and cold crystallization, Δ*H*_c_ and Δ*H*_cc_, CF and CF_cc_, respectively, melting temperature, *T*_m_, and enthalpy, Δ*H*_m_.

		Melt–Fast Cooled *Scan* 1						Melt–Slower Cooling *Scan* 2					
Sample	*M*_n_ (g/mol)	*T*_g_ (°C)	Δ*c*_p_ (J/g∙K)	*T*_cc_ (°C)	CF_cc_ (wt)	*T*_m_ (°C)	CF_m_ * (wt)	*T*_c_ (°C)	CF_c_ (wt)	*T*_g_ (°C)	Δ*c*_p_ (J/g∙K)	*T*_m_ (°C)	Δ*H*_m_ (J/g)
PEF neat	8.9 k	82	0.46	174	0.05	203	0.06	-	0	82	0.46	-	0
PEF–bioglass	10.6 k	84	0.38	-	0	-	0	-	0	84	0.38	-	0
PEF–ZnO	7.4 k	74	0.48	155	0.01	200	0.01	-	0	74	0.46	199	0.3
PEF–ZrO_2_	9.6 k	82	0.46	N/A	N/A	205	0.002	-	0	82	0.46	-	0

(*) CF is estimated by comparing the corresponding Δ*H* with the theoretical value for the heat of fusion of neat 100% crystalline PEF, i.e., 137 J/g.

**Table 4:** Thermal characteristics from the DSC scan after annealing (160 °C, 1 h).

Samples	T_m1_ (°C)	T_m2_ (°C)	ΔH_m_ (J/g)	*Xc* ^a^ (%)	*Xc* ^b^ (%)
PEF neat	179	201	40.1	29	33
PEF–bioglass	173	207	37.5	29	31
PEF–ZnO	172	202	47.6	35	41
PEF–ZrO_2_	172	202	33.1	24	32

^a^ From DSC data, using Equation (3). ^b^ From XRD data, using Equation (4).

**Table 5:** The estimated dielectric glass transition temperature, *T*_g_._diel_, and fragility index for the *α* relaxation, *m*_α_. The calorimetric data for *T*_g_ in the amorphous state are shown for comparison.

		DSC *Scan* 1	BDS *Melted and Fast-Cooled*	
Sample	*M_n_* (g/mol)	*T_g_* (°C)	*T_g, diel_* (°C)	Fragility Index m_α_
PEF neat	8.9 k	82	75	100
PEF–bioglass	10.6 k	84	78	102
PEF–ZnO	7.4 k	74	72	101
PEF–ZrO_2_	9.6 k	82	77	103

## Data Availability

Data are contained within the article and Supplementary Materials.

## Supplementary Materials

The following supporting information can be downloaded at: https://www.mdpi.com/article/10.3390/polym15234502/s1, Figure S1: The time-temperature profiles were employed for the DSC measurements during scans 1 and 2. Figure S2: (a) N_2_ adsorption-desorption isotherm and (b) respective pore size distribution of the bioglass. Figure S3: SEM micrographs of Ce-bioglass nanoparticles. Figure S4: PXRD patterns of Ce-bioglass (the intensity below 2 deg is due to the main beam). References [47,48,49,50,51,52,53] are cited in the supplementary materials.
