# Supplementary material for: Synthesis of Poly(ethylene furanoate) Based Nanocomposites by In Situ Polymerization with Enhanced Antibacterial Properties for Food Packaging Applications
Source: Polymers (Basel). 2023 Nov 23;15(23):4502. doi: 10.3390/polym15234502 (PMC10708257; doi:10.3390/polym15234502)
Supplement: Supplementary file 1 [file polymers-15-04502-s001.zip › polymers-2687143-supplementary.pdf]

polymers MDPI

Supplementary Materials

Johan Stanley [1], Eleftheria Xanthopoulou [1], Matjaž Finšgar [2], Lidija Fras Zemljič [3], Panagiotis A. Klonos [4], Apostolos Kyritsis [4], Savvas Koltsakidis [5], Dimitrios Tzetzis [5], Dimitra A. Lambropoulou [6,7], Diana Baciu [8], Theodore A. Steriotis [8], Georgia Charalambopoulou [8] and Dimitrios N. Bikiaris [1,*]

[1] Laboratory of Chemistry and Technology of Polymers and Colors, Department of Chemistry, Aristotle University of Thessaloniki, GR-54124 Thessaloniki, Greece; johansta@chem.auth.gr (J.S.); elefthxanthopoulou@gmail.com (E.X.)
[2] Faculty of Chemistry and Chemical Engineering, University of Maribor, SI-2000 Maribor, Slovenia; matjaz.finsgar@um.si
[3] Faculty of Mechanical Engineering, University of Maribor, SI-2000 Maribor, Slovenia; lidija.fras@um.si
[4] Department of Physics, National Technical University of Athens, Zografou Campus, GR-15780 Athens, Greece; pklonos@central.ntua.gr (P.A.K.); akyrits@central.ntua.gr (A.K.)
[5] Digital Manufacturing and Materials Characterization Laboratory, International Hellenic University, GR-57001 Thessaloniki, Greece; skoltsakidis@ihu.edu.gr (S.K.); d.tzetzis@ihu.edu.gr (D.T.)
[6] Laboratory of Environmental Pollution Control, Department of Chemistry, Aristotle University of Thessaloniki, GR-54124 Thessaloniki, Greece; dlambro@chem.auth.gr
[7] Center for Interdisciplinary Research and Innovation (CIRI-AUTH), Balkan Center, GR-57001 Thessaloniki, Greece
[8] National Center for Scientific Research "Demokritos", GR-15341 Ag. Paraskevi Attikis, Greece; dianabaciuro@yahoo.com (D.B.); t.steriotis@inn.demokritos.gr (T.A.S.); gchar@ipta.demokritos.gr (G.C.)
* Correspondence: dbic@chem.auth.gr

S1. Materials Characterization

S1.1. Characterization of Mesoporous Bioglass (Ce-bioglass)

S1.1.1. SEM Analysis

Scanning Electron Microscope (SEM) images of the bioglass material was obtained using a Jeol JSM 7401F Field Emission scanning electron microscope coupled with an energy dispersive X-ray analyzer (EDX) for element analysis. All specimens were coated with carbon black to avoid charging under the electron beam.

S1.1.2. Fourier-Transform Infrared Spectroscopy (FTIR)

Infrared (IR) spectra were obtained using a Thermo Scientific Nicolet 6700 FTIR equipped with a N_2 purging system and a LN2-cooled wide range Mercuric Cadmium Telluride detector in the mid-infrared region of 4000–400 cm^{-1}.

S1.1.3. Powder X-Ray Diffraction (PXRD)

PXRD patterns of the bioglass were recorded on a Rigaku R-AXIS IV Imaging Plate Detector mounted on a Rigaku RU-H3R Rotating Copper Anode X-ray Generator (λ=0.154 nm).

S1.1.4. Pore Properties

The pore properties of the bioglass were determined by nitrogen adsorption/desorption measurements at 77 K using a volumetric gas adsorption analyser (AUTOSORB-1-MP, Quantachrome Instruments, Boynton Beach, FL, USA). Prior to measurement, the sample was appropriately outgassed (at 250 °C for 12 h) under ultra-high vacuum (10^{-6} mbar), while ultra-pure N_2 was used. The respective BET area value was calculated by the Brunauer-Emmett-Teller method, following the BET consistency criteria and the pore size distributions was deduced by fitting the adsorption isotherms on the basis of a non-local density functional theory (NLDFT) kernel developed for N_2 at 77K on silica materials with cylindrical pores.

S1.2. Differential Scanning Calorimetry (DSC)

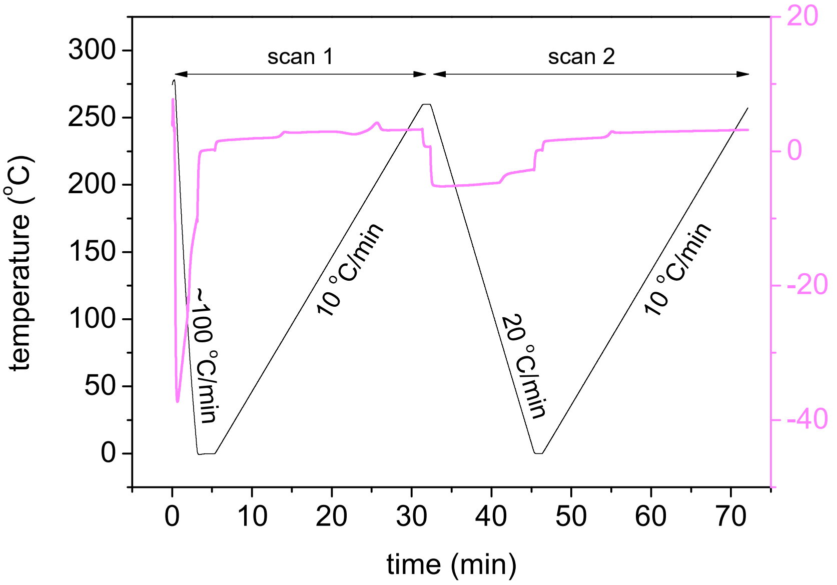

Figure S1. The time-temperature profiles were employed for the DSC measurements during scans 1 and 2**.**

S.2. Results and Discussion

S.2.1. Pore size Distribution of Prepared Bioglass

As shown in Figure S2, the N_2 adsorption/desorption isotherm at 77 K of the calcined Ce-bioglass is of type IV (according to IUPAC classification), characteristic of mesoporous materials. The sample exhibited a BET area of 730 m^2/g, mean pore size around 3 nm and a total pore volume of 0.71 cm^3/g, as illustrated in Table S1.

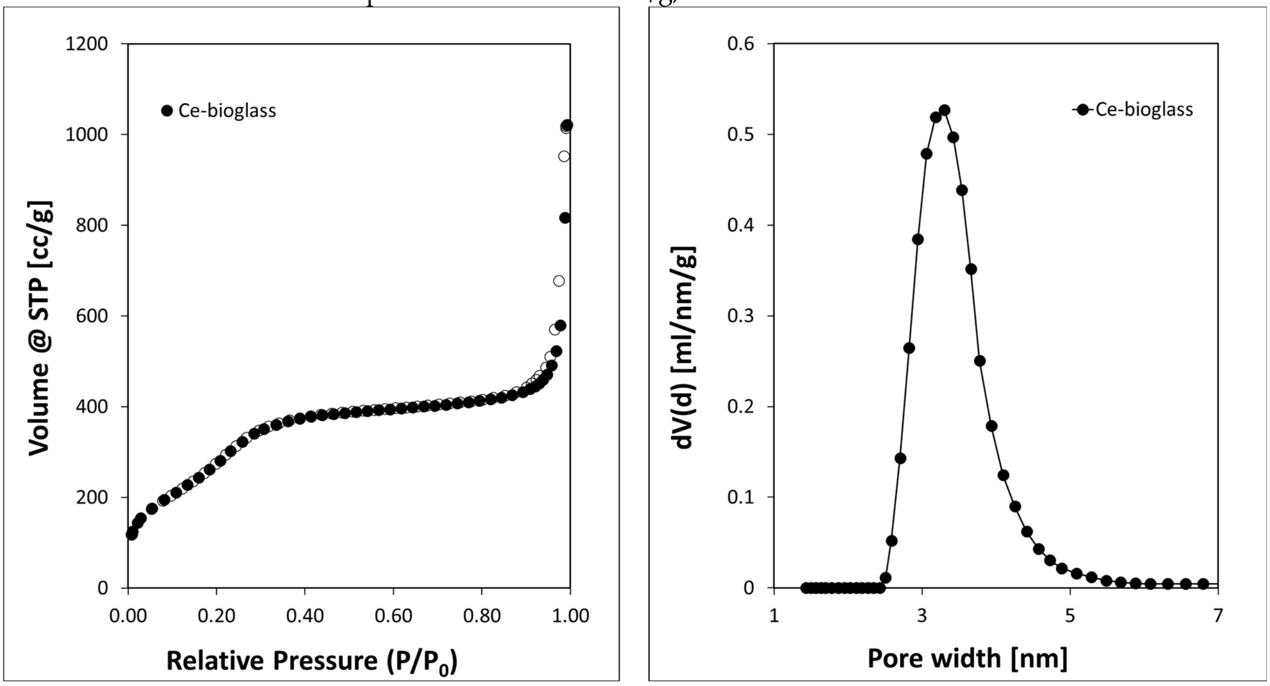

(**a**) (**b**)

Figure S2. (**a**) N_2 adsorption-desorption isotherm and (**b**) respective pore size distribution of the bioglass.

Table S1. Nominal compositions (mol %) and textural properties of the prepared bioactive glasses

Ce-Bioglass (SiO_2-SrO -P_2O_5-MgO-CeO_2) (mol.%)	Sample Name	Surface Area (m^2/g)	Particle Size Diameter (nm)	Pore Size (nm)	Total Pore Volume (cm^3/g)
78.5SiO_2-10SrO-10P_2O_5-0.5MgO-1CeO_2 (mol.%)	Ce-bioglass	730	100-150	3.17	0.71

S.2.2. Scanning Electron Microscope (SEM)

SEM analysis of the bioglass showed that the sol-gel method employed and the calcination at 600 °C leads to aggregated nanoparticles with diameter between 100-150 nm as shown in Figure S3.

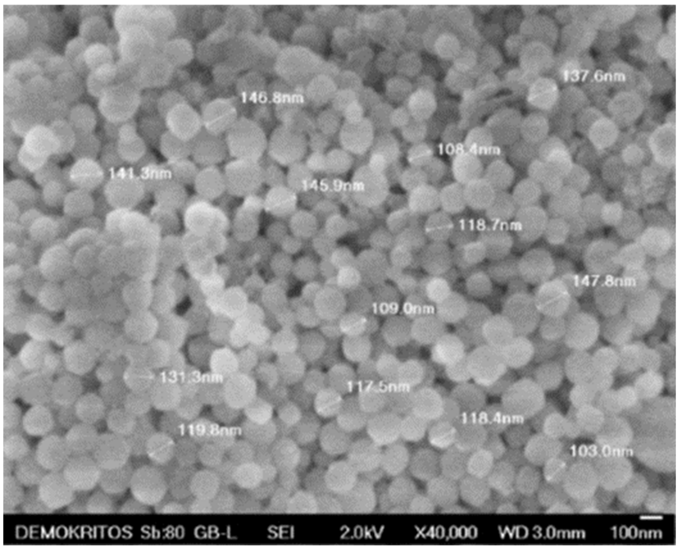

Figure S3. SEM micrographs of Ce-bioglass nanoparticles.

S.2.3. Powder X-Ray Diffraction (PXRD)

The PXRD pattern (Figure S4) of the Ce-bioglass shows small angle diffraction peaks at 2θ ca. 2.9, and 4.8° indicating that the pores exhibit long range ordering [1,2], while in the wide-angle region some small and broad peaks are observed that could be associated with the presence of strontium crystalline compounds such as strontium carbonate ($SrCO_3$, JCPDS No. 05-0418) and strontium silicate (Sr_2SiO_4, JCPDS No. 38-0271) [3,4].

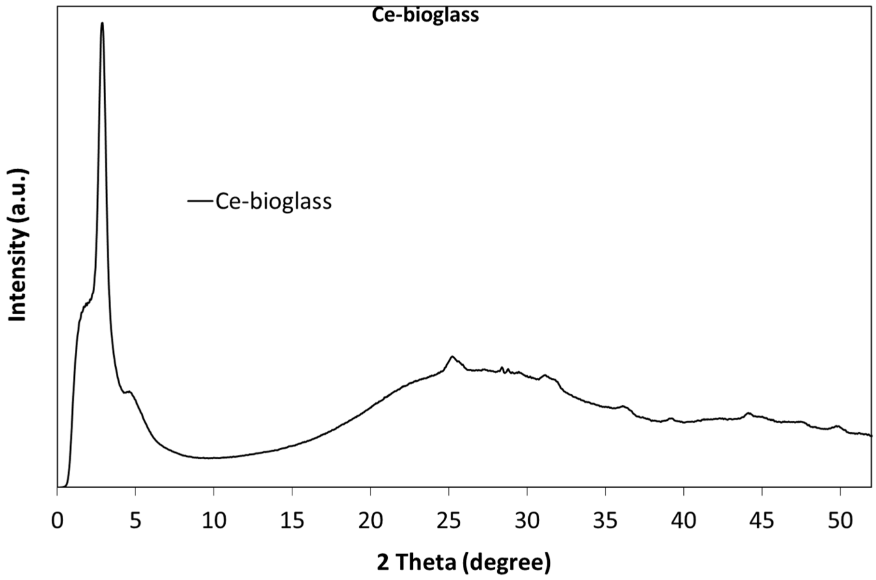

Figure S4. PXRD patterns of Ce-bioglass (the intensity below 2 deg is due to the main beam).

S.2.4. Fourier-transform infrared spectroscopy (FTIR)

Figure S5 presents the IR spectra obtained for the bioglass exhibiting the stretching and bending vibrations of the Si–O–Si bridge in both cases. Characteristic bands between 400 and 500 cm^{-1} are assigned to the bending vibrations of the Si–O–Si and O–Si–O bonds; the peak in the 760–810 cm^{-1} range corresponds to the stretching vibrations of the O–Si–O bonds; the peak in the 1000–1100 cm^{-1} range may be attributed to the symmetric stretching vibration of the Si–O–Si bonds [5,6]; the absorption peak at around 1630 cm^{-1} is associated with the stretching mode of the OH group. In addition, there are no peaks that can be assigned to organic matter, confirming the purity of the materials. The two additional peaks around 1470 cm^{-1} and 840 cm^{-1} are attributed to C–O stretching in carbonate groups (CO_3^{2-}) that can be formed by the reaction of the bioglass with the atmospheric CO_2 [6,7].

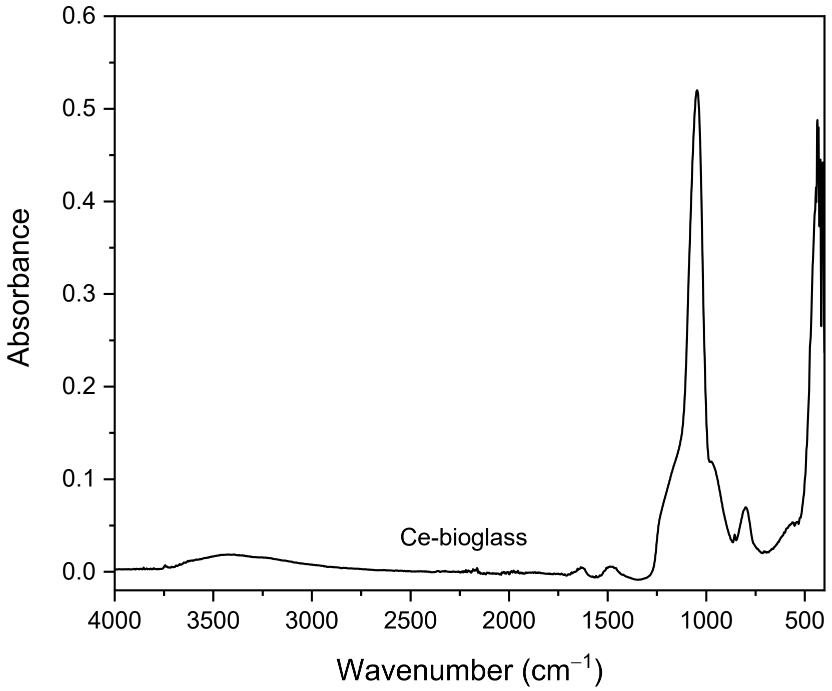

Figure S5. FTIR spectra of Ce-bioglass.

Author Contributions: Conceptualization, methodology, formal analysis, investigation, J.S., M.F., P.A.K., L.F.Z., E.X., S.K., D.T., D.B., T.A.S., A.K. and D.N.B.; writing—original draft preparation, J.S., P.A.K., D.T. and D.N.B.; writing—review and editing, J.S., E.X., P.A.K., D.T., G.C., L.F.Z. and D.N.B.; supervision, D.N.B., L.F.Z. and D.A.L.; project administration, D.N.B., L.F.Z. and D.A.L.; funding acquisition, D.N.B., L.F.Z. and D.A.L. All authors have read and agreed to the published version of the manuscript.

Funding: This research is funded under the project Advanced Research and Training Network in Food Quality, Safety, and Security—FoodTraNet—H2020-MSCA-ITN-2020. The authors acknowledge the Slovenian Research Agency (grant P2-0118). The project is cofinanced by the Republic of Slovenia, the Ministry of Education, Science and Sport, and the European Union under the European Regional Development Fund.

Institutional Review Board Statement: Not applicable.

Data Availability Statement: Data are contained within the article and supplementary materials.

Acknowledgments: We thank Nikolaidis Nikolaos, Laboratory of Polymer and Color Chemistry and Technology, Aristotle University of Thessaloniki (AUTh) for his help in color calorimeter measurements. We thank Eleni Pavlidou and Chrysanthi Papoulia, School of Physics, AUTh for the SEM measurements. Also, we thank Tanja Kos, Laboratory for Characterization and Processing of Polymers, University of Maribor for her help in contact angle measurements and antioxidant analysis.

Conflicts of Interest: The authors declare no conflicts of interest.

References

1. Arcos, D.; Vila, M.; López-Noriega, A.; Rossignol, F.; Champion, E.; Oliveira, F.J.; Vallet-Regí, M. Mesoporous Bioactive Glasses: Mechanical Reinforcement by Means of a Biomimetic Process. *Acta Biomater.* **2011**, *7*, 2952–2959. https://doi.org/10.1016/j.actbio.2011.02.012.
2. Baino, F.; Fiorilli, S.; Mortera, R.; Onida, B.; Saino, E.; Visai, L.; Verné, E.; Vitale-Brovarone, C. Mesoporous Bioactive Glass as a Multifunctional System for Bone Regeneration and Controlled Drug Release. *J. Appl. Biomater. Funct. Mater.* **2012**, *10*, 11–20. https://doi.org/10.5301/JABFM.2012.9270.
3. Solgi, S.; Khakbiz, M.; Shahrezaee, M.; Zamanian, A.; Tahriri, M.; Keshtkari, S.; Raz, M.; Khoshroo, K.; Moghadas, S.; Rajabnejad, A. Synthesis, Characterization and In Vitro Biological Evaluation of Sol-Gel Derived Sr-Containing Nano Bioactive Glass. *Silicon* **2017**, *9*, 535–542. https://doi.org/10.1007/s12633-015-9291-x.
4. Terzopoulou, Z.; Baciu, D.; Gounari, E.; Steriotis, T.; Charalambopoulou, G.; Tzetzis, D.; Bikiaris, D. Bisphosphonate-Loaded Bioactive Glasses for Potential Bone Tissue Engineering Applications. *Molecules* **2019,** *7*, 3067. https://doi.org/10.3390/molecules24173067.
5. Saravanapavan, P.; Hench, L.L. Mesoporous Calcium Silicate Glasses. I. Synthesis. *J. Non. Cryst. Solids* **2003**, *318*, 1–13. https://doi.org/10.1016/S0022-3093(02)01864-1.
6. Moghanian, A.; Firoozi, S.; Tahriri, M. Characterization, in Vitro Bioactivity and Biological Studies of Sol-Gel Synthesized SrO Substituted 58S Bioactive Glass. *Ceram. Int.* **2017**, *43*, 14880–14890. https://doi.org/10.1016/j.ceramint.2017.08.004.
7. Łączka, M.; Cholewa-Kowalska, K.; Osyczka, A.M. Bioactivity and Osteoinductivity of Glasses and Glassceramics and Their Material Determinants. *Ceram. Int.* **2016**, *42*, 14313–14325. https://doi.org/10.1016/j.ceramint.2016.06.077.

Disclaimer/Publisher's Note: The statements, opinions and data contained in all publications are solely those of the individual author(s) and contributor(s) and not of MDPI and/or the editor(s). MDPI and/or the editor(s) disclaim responsibility for any injury to people or property resulting from any ideas, methods, instructions or products referred to in the content.
